# Maternal health status and household food security on determining childhood anemia in Bangladesh -a nationwide cross-sectional study

**DOI:** 10.1186/s12889-021-11581-3

**Published:** 2021-08-21

**Authors:** Masum Ali, Md. Ruhul Amin, Johan Jarl, Nick Chisholm, Sanjib Saha

**Affiliations:** 1 Department of Food Business and Development, O’Rahilly Building, University College Cork, Cork, Ireland; 2 Institute of Nutrition and Food Science (INFS), University of Dhaka, Dhaka, 1000 Bangladesh; 3 Department of Clinical Science (Malmö), Health Economics Unit, Lund University, Medicon Village, Scheelevagen 2, SE-223 63 Lund, Sweden; 4 Center for Global Development and Department of Food Business and Development, O’Rahilly Building, University College Cork, Cork, Ireland

**Keywords:** anemia, Child health, Maternal health, Demography and health survey, Bangladesh

## Abstract

**Background:**

The aim of this study was to examine the effect of household food security on childhood anemia in Bangladesh while controlling for socioeconomic and demographic factors.

**Methods:**

We used nationally representative Bangladesh Demographic Health Survey (BDHS) 2011 data for this study, the only existing survey including anemia information and household food security. The sample included 2171 children aged 6–59 months and their mothers. Differences between socioeconomic and demographic variables were analyzed using Chi-square test. Univariate and multivariate logistic regression analyses were performed to estimate the effects of different socioeconomic and demographic factors on childhood anemia. We also performed mediation analysis to examine the direct and indirect effect of household food security on childhood anemia.

**Results:**

In Bangladesh, 53% male (95% CI: 50–56) and 51% female (95% CI: 47–54) children aged 6–59 months were anemic in 2011. The food insecure households have 1.20 times odds (95% CI: 0.97–1.48) of having anemic children comparing to food secure households in the unadjusted model. On the other hand, anemic mothers have 2 times odds (95% CI: 1.67–2.44) of having anemic children comparing to non-anemic mothers. However, household food security is no longer significantly associated with childhood anemia in the adjusted model while mothers’ anemia remained a significant factor (OR 1.87: 95% CI: 1.53–2.29). Age of children is the highest associated factor, and the odds are 4.89 (95% CI: 3.21–7.45) for 6–12 months old children comparing to 49–59 months in the adjusted model. Stunting and household wealth are also a significant factor for childhood anemia. Although food security has no significant direct effect on childhood anemia, maternal anemia and childhood stunting mediated that relationship.

**Conclusions:**

Future public health policies need to focus on improving mothers’ health with focusing on household food security to eliminate childhood anemia.

**Supplementary Information:**

The online version contains supplementary material available at 10.1186/s12889-021-11581-3.

## Background

Anemia is one of the most common and widespread disorder that especially affect young children and women of reproductive age. Global anemia prevalence is estimated at 38% in pregnant women (aged 15–49 years), 29% in non-pregnant women (aged 15–49 years) and 42.6% in preschool children (aged 6–59 months) [1] Iron deficiency accounts for half of the world’s anemia burden with 97% occurring in the low- and middle-income countries [1] Iron deficiency anemia caused 8.8% of the total disability globally and children and women are bearing the highest burden [2]. In terms of monetary value, the median annual economic loss in 10 developing countries was estimated at US$16.78 per capita which is equal to 4% of the gross domestic products in these countries [3].

In children, anemia negatively affects (potentially irreversibly) the central nervous system, the immune system, cognitive development, physical growth, long term neurodevelopment, and behavior [4]. The most common form of anemia is iron deficiency anemia which is caused by poor nutritional intake of iron and low iron bioavailability. Epidemiological research suggested that factors associated with childhood anemia are complex and multidimensional which may involve nutritional, socioeconomic, environmental, biological and cultural characteristics [5], but exposure to a single factor might be sufficient for a child to develop anemia. Maternal health status has been shown to be an important factor for childhood anemia [6, 7]. Maternal anemia is associated with a 4-fold risk of developing childhood anemia comparing to mother without anemia [6], while both maternal under-and overweight have been shown to be associated with childhood anemia.

Food insecurity adversely influences the dietary quality of households, particularly nutrients rich food intake [8]. Food insecure households have less diverse diet which increases the risk of anemia for children as well as adult women [9]. Infants living in food insecure households are 1.42 times likely to be anemic at age 18 months [10]. In Bangladesh, 40 million people are food insecure, and 11 million people are suffering from acute food shortage [11] and it is unlikely that Bangladesh will ensure food security for the full population by 2021, despite several national plans of actions. According to the Global Hunger Index, Bangladesh ranks 75th out of 107 countries with a score of 20.4 in 2020. This means the level of hunger is “serious” in Bangladesh [12] and achieving the Sustainable Development Goal 2 (End hunger, achieve food security and improved nutrition and promote sustainable agriculture) by 2030 will be difficult [13]. Furthermore, micronutrient deficiencies are common, especially vitamin A, iron, and the micronutrient supplementation are inadequate [14]. For example, 42% women of aged 15–49 are anemic and the rate is even higher for pregnant women (50%) [15]. The prevalence of anemia among children aged 6–59 months in Bangladesh increased to 64% in 2004 from 47% in 2001 [16]. Moreover, infants at 6 months from rural areas are unexpectedly more anemic (71.9%) [17]. The National Micronutrient Survey (2013), using a different methodology from previous studies, reports that about 33% of children aged 6–59 months are anemic [18]. Thus, the World Health Organization has declared childhood anemia as a severe public health problem for Bangladesh [1].

In a meta-analysis, a positive relationship between food insecurity and anemia was shown where infants, toddlers and adult women has the highest risk of anemia in food insecure households [19]. Household food insecurity is significantly associated with childhood anemia in other developing countries such as Mexico [20] and Ethiopia [6]. Several studies have tried to determine which factors are the most important for the development of childhood anemia in Bangladesh [17, 21]. Eneroth et al. found that infant anemia is associated with infection, low birth weight and iron deficiency [21]. However, the study was conducted in a specific area with a sample of 580 six-month old infants. Another study on 6 to 12 months old infants found that anemia was significantly associated with sex and age of the children, maternal short stature, and child nutritional status, but not with maternal education, household size or household expenditure [22]. Shakur et al. found that birth weight and month of birth have a significant association with children’s hemoglobin status [17]. Several studies have identified the determinants of childhood anemia based on nationally representative data from the Bangladesh Demography and Health Survey’2011 [23–26]. However, none of the studies have considered the impact of household food security on childhood anemia. Furthermore, these studies did not investigate the effect of maternal anemia and maternal body mass index (BMI) on childhood anemia. Only one study has considered the effect of household food security on maternal anemia but not on childhood anemia [27].

Our hypothesis is that household food security is directly or indirectly associated with childhood anemia in Bangladesh. Therefore, the objective of this study is 1) to investigate the association between food security and anemia in 6 to 59 months old children in Bangladesh and 2) to examine the extent to which other factors such as maternal anemia or household wealth explain these relationships.

## Methods

### Data source

This study was a cross-sectional analysis of the Bangladesh Demographic and Health Survey 2011 (BDHS’2011) dataset. The BDHS’2011 was conducted by the National Institute for Population Research and Training (NIPORT) of the Ministry of Health and Family Welfare in Bangladesh in the second half of 2011 [15]. Later versions of the BDHS survey (2014 and 2018) does not include information on maternal/child anemia as well as household food security and can therefore not be used for the current study.

**Fig. 1:**
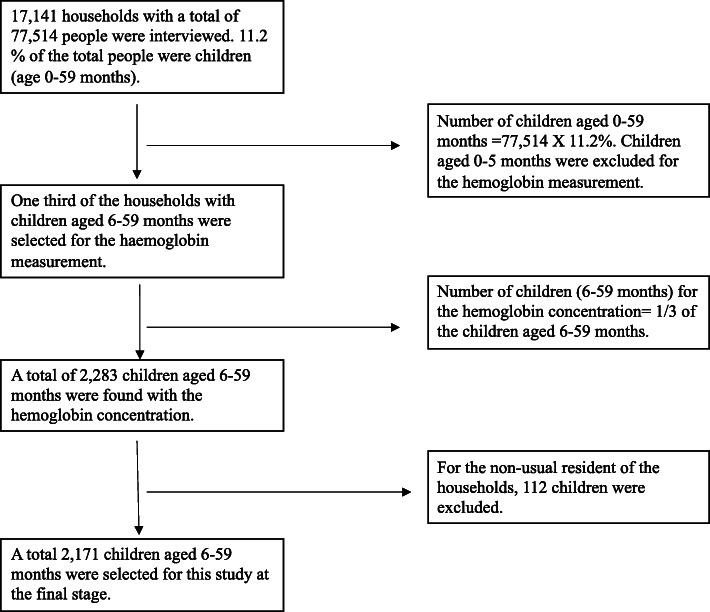
Sample size selection procedure

BDHS’11 is a nationally representative survey with a stratified, multistage cluster sample of 600 enumeration areas (EAs), 207 from the urban area and 393 from the rural area. A systematic sample of 30 households on average was selected from each EA that led to 17,964 households. Among them, a total of 17,141 households were interviewed successfully. From this sample, a subsample of one-third of the households has been selected for hemoglobin measurement of children aged 6–59 months which leads to 2283 children. From these, 112 children were removed due to non-usual resident leaving a sample size for this study of 2171 children. Details of data collection and management procedures are described elsewhere [15]. In Fig. 1, we present the sample size selection procedure.

### Variable specification

#### Dependent variable

Anemia is characterized by low level of hemoglobin (Hb) in the blood. The BDHS’2011 used HemoCure rapid testing methodology to measure Hb. The details of blood collection procedure have been described elsewhere [15]. The Hb level was adjusted for altitude in BDHS’2011 by using formulas from Center for Disease Control [15]. Anemia status in children was categorized by level of Hb in blood where mild anemia was 10.0 to 10.9 g/dl, moderate anemia was 7.0 to 9.9 g/dl, and severe anemia was less than 7.0 g/dl following the cut-off from the World Health Organization [28]. For further analyses, we combined mild, moderate and severe anemia into anemia (Hb < 11 g/dl) and thus childhood anemia was dichotomized as “anemia” and “no anemia”.

#### Independent variables

For the first time in Bangladesh, information regarding food security was included in BDHS’2011. An operational definition of household food security is that a household is food secure when “it has access to the food needed for a healthy life for all its members (adequate in terms of quality, quantity, safety and culturally acceptable), and when it is not at undue risk of losing such access” (UN ACC/SCN 1991). In BDHS’2011, food security was measured by five questions regarding the availability of food and then converted to Household Food Insecurity Access Scale (HFIAS) as suggested by Coates et al. [29]. The questions are: 1) How often did you eat three square meals’ (full stomach meals) a day in the past 12 months (not a festival day), 2) In the last 12 months how often did you yourself skip entire meals because there was not enough food, 3) In the last 12 months how often did you personally eat less food in a meal because there was not enough food? 4) In the last 12 months, how often did you or any of your family have to eat wheat (or another grain) although you wanted to eat rice (not including when you were sick), 5) In the past 12 months how often did your family have to ask food from relatives or neighbors to make a meal? The details of the questionnaire and scale can be found elsewhere [15]. For this study, we termed a household as food secure if women did not experience any food insecurity (access) or had not worry about food in the last 12 months. If this condition was not fulfilled, the household was deemed to be food insecure.

Mothers’ health status was considered using two variables: BMI and anemia level of the mother. The mother’s BMI was measured as weight in kilograms divided by height in meters squared (kg/m2). Mother’s BMI was categorized as overweight/underweight and normal weight. BMI of less than 18.5 kg/m2 was categorized as underweight and higher than 25 was overweight, whereas in between was normal weight following the cut-off from WHO [30]. Anemia of women was measured by the same method as children except the capillary blood was exclusively collected from a finger prick. The Hb level was adjusted for pregnancy status and altitude. Women were considered to have anemia if Hb was lower than 12 g/dl for non-pregnant and 11 g/dl for pregnant women [28].

The variable currently breastfeeding comprised if the women were breastfeeding any children at the time of the survey. Sex and age of the children in months were included. Age of the children was categorized in 5 groups where the first group had children aged 6 to 11 months with 12 months intervals for the following groups. Child undernutrition was calculated following the cut-off from the WHO [31]. Children below two standard deviation (SD) height for age were considered stunted. Education level of mother and their husband was categorized into low education, which combines no education, primary education, secondary education and high education as education higher than secondary education. Primary and secondary education are defined as completing grades 5 and 10 respectively. Religion was dichotomized into Muslim and Hindu and others as the major religion of Bangladesh is Islam. Bangladesh has seven administrative regions; Barisal, Chittagong, Dhaka, Khulna, Rajshahi, Rangpur and Sylhet that differ in terms of geographical appearances i.e., hilly area, flood-prone area etc. which may have a role on household food security. A relative index of household wealth based on interviewer-observed assets, consumer items, and dwelling was created based on factor analysis and then categorized into five quintiles by BDHS’2011 (poorest, poorer, middle, richer and richest). We dichotomized the variable by merging the two poorest quintiles into a poor category and the three highest quintiles into a non-poor category.

### Data analysis

We have adjusted for the complex survey design, as outlined above, by applying weights developed by the BDHS’2011. We performed chi-square test to check the differences between dependent and independent variables. In the univariable analyses, we estimated the effect of the independent variables on childhood anemia by logistic regression analysis. We also used multivariate logistics regression adapting the conceptual model proposed by Siva et al. [32], using three Blocks. In Block 1 (socioeconomic characteristics), we included household food security, wealth status, religion, region, and father’s education. In Block 2 (maternal characteristics), we included maternal anemia, BMI and education. In Block 3 (child characteristics), we included child sex, age, breastfeeding status, and undernutrition.

**Fig. 2:**
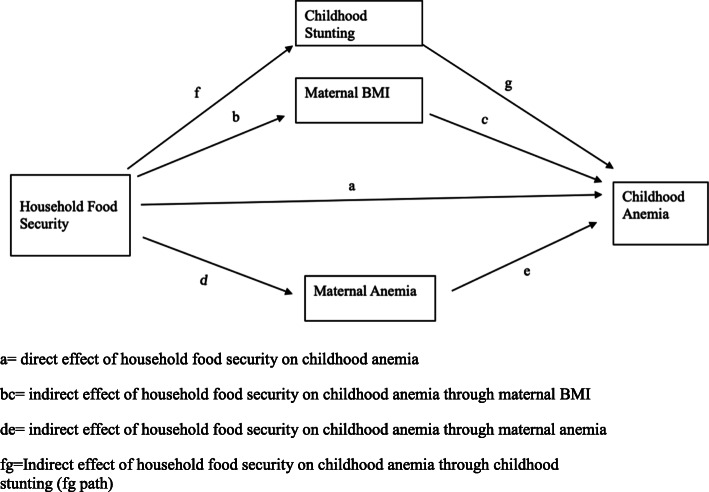
Mediation analysis of household food security on childhood anemia. a = direct effect of household food security on childhood anemia. bc = indirect effect of household food security on childhood anemia through maternal BMI. de = indirect effect of household food security on childhood anemia through maternal anemia. fg = Indirect effect of household food security on childhood anemia through childhood stunting (fg path)

The crude and adjusted odds ratios (OR) are presented with 95% confidence interval (CI). We evaluated multicollinearity for the models by checking correlation and variance inflation factors (VIF) and tolerance values [33]. A mediation analysis was performed to examine the indirect effect of household food security on childhood anemia through maternal BMI, maternal anemia, and childhood stunting [34]. We present the mediation pathway in Fig. 2.

## Results

In Table 1 we show the distribution of anemia among 6- to 59-month-old children for different independent variables. Except for sex and maternal BMI, all included variables are significantly associated with prevalence of anemia.

**Table 1:** Percentage of anemia among the 6 to 59-month-old children in relation to different socioeconomic variables (*n* = 2171)

Variables	Number (%)	Anemia,	***p***-value^**a**^
		Number (%)	
**Household Food Security**			
Food secure	1323 (61)	664 (50)	0.09
Food insecure	843 (39)	461 (55)	
**Wealth index**			
Poor	989 (46)	569 (58)	0.00
Non-poor	1177 (54)	556 (47)	
**Religion**			
Muslim	1991 (92)	1014 (51)	0.00
Hindu and others	176 (8)	111 (63)	
**Region**			
Barisal	118 (5)	71 (60)	0.07
Chittagong	480 (22)	252 (52)	
Dhaka	665 (31)	318 (48)	
Khulna	203 (9)	111 (55)	
Rajshahi	276 (13)	138 (50)	
Rangpur	249 (12)	147 (59)	
Sylhet	176 (8)	89 (50)	
**Father’s education**			
No or low education	1907 (88)	1016 (53)	0.00
Higher education	258 (12)	107 (42)	
**Mother’s BMI**			
Normal Weight	1270 (59)	646 (51)	0.33
Over/under weight	888 (41)	473 (53)	
**Mother’s anemia**			
Not anemic	1192 (56)	528 (44)	0.00
Anemic	941 (44)	579 (62)	
**Mother’s education**			
No or low education	2023 (93)	1072 (53)	0.00
Higher education	148 (7)	53 (37)	
**Sex of children**			
Male	1105 (51)	586 (53)	0.36
Female	1062 (49)	539 (51)	
**Age of the children**			
6–12	288 (13)	191 (73)	0.00
13–24	454 (21)	342 (70)	
25–36	426 (20)	212 (48)	
37–47	523 (24)	229 (42)	
49–59	475 (22)	206 (38)	
**Currently Breastfeeding**			
Yes	1385 (64)	811 (59)	0.00
No	782 (36)	314 (40)	
**Child nutritional status**			
Normal	1229 (58)	604 (49)	0.03
Stunted	893 (42)	491 (55)	

^a^*P* is the chi-squared test

In Table 2 we present the unadjusted and adjusted odd ratios of socioeconomic, maternal and child characteristics variables on childhood anemia. Age of the children was the strongest factor (OR: 4.57; 95% CI: 3.17–6.60) for 6–12 months old children compared to 49–59 months in the unadjusted model. Stunting of the children was a significant factor (OR:1.27; 95% CI: 1.03–1.56) for the childhood anemia. Food insecure households have 1.20 times odds (95% CI: 0.97–1.48) of having anemic child compared to food secure household in the unadjusted model. Anemic mother has 2 times higher odds (OR: 2.02; 95% CI: 1.67–2.44) of having childhood anemia compared to the non-anemic mother. Mother’s and father’s education are also significant factors of childhood anemia together with the current breastfeeding status of the mother.

**Table 2:** Unadjusted and Adjusted logistic regression analyses of the odds of childhood anemia

	Unadjusted OR (CI)	Block 1 (Socio-economic characteristics) AOR (CI)	Block 2 (Maternal characteristics) AOR (CI)	Block 3 (Child characteristics) AOR (CI)	Final Model AOR (CI)
**Household Food Security**					
Food secure®	1	1			1
Food insecure	1.20* (0.97–1.48)	1.04 (0.83–1.30)			1.05 (0.83–1.34)
**Wealth index**					
Poor	1.51*** (1.25–1.84)	1.39*** (1.13–1.73)			1.28** (1.01–1.61)
Non-poor®	1	1			1
**Religion**					
Muslim	0.61*** (0.44–0.83)	0.61*** (0.45–0.83)			0.61*** (0.43–0.88)
Hindu and others®	1	1			1
**Region**					
Barisal	1.50** (1.06–2.13)	1.51** (1.06–2.15)			1.79*** (1.23–2.59)
Chittagong	1.09 (0.76–1.56)	1.18 (0.83–1.68)			1.11 (0.76–1.62)
Dhaka	0.90 (0.66–1.24)	0.98 (0.72–1.33)			0.97 (0.69–1.36)
Khulna	1.18 (0.84–1.66)	1.28 (0.91–1.79)			1.53** (1.03–2.25)
Rajshahi	0.99 (0.71–1.38)	1.02 (0.73–1.42)			1.13 (0.76–1.68)
Rangpur	1.41** (1.01–1.98)	1.32 (0.95–1.83)			1.49** (1.04–2.13)
Sylhet®	1				1
**Father’s education**					
No or low education	1.61*** (1.20–2.15)	1.38** (1.03–1.85)			1.29 (0.95–1.75)
High education®	1	1			1
**Mother’s BMI**					
Under/over weight	1.10 (0.91–1.34)		1.11 (0.91–1.35)		1.06 (0.86–1.31)
Normal®	1		1		1
**Mother’s anemia**					
Anemic	2.02*** (1.67–2.44)		1.97*** (1.63–2.39)		1.87*** (1.53–2.29)
Not anemic®	1		1		1
**Mother’s education**					
No or low education	1.92*** (1.29–2.86)		1.75*** (1.16–2.63)		1.36 (0.85–2.19)
High education®	1		1		1
**Sex of children**					
Male	1.09 (0.90–1.33)			1.11 (0.90–1.36)	1.18 (0.95–1.46)
Female®	1			1	1
**Age of the children**					
6–12	4.57*** (3.17–6.60)			4.26*** (2.87–6.34)	4.89*** (3.21–7.45)
13–24	3.44*** (2.50–4.73)			2.99*** (2.12–4.22)	3.59*** (2.52–5.11)
25–36	1.57*** (1.17–2.10)			1.40** (1.03–1.90)	1.58*** (1.15–2.16)
37–48	1.11 (0.83–1.48)			1.08 (0.80–1.44)	1.19 (0.89–1.59)
49–59	1			1	1
**Currently Breastfeeding**					
Yes	2.11*** (1.71–2.59)			1.21 (0.95–1.54)	1.01 (0.78–1.31)
No®	1			1	1
**Child Nutritional status**					
Stunted	1.27** (1.03–1.56)			1.40*** (1.13–1.74)	1.33** (1.06–1.65)
Normal®	1			1	1

Note. *OR* Odd Ratio, *AOR* Adjusted Odd Ratio

*p < 0.05; **p < 0.01; ***p < 0.001

It is interesting to note that household food security is no longer a significant factor of childhood anemia when controlled for other socioeconomic variables (Block 1, Adjusted model). Anemic mothers have around 2 times odds of having anemic children compared non-anemic mother in the adjusted models (Block 2). Currently breastfeeding is also no longer a significant factor in the adjusted models but it was significant in Block 3. The age of the child remains the biggest significant factor after adjusting for other socioeconomic variables in the adjusted models (Block 3). Stunting is a significant factor adjusted model (Block 3). Wealth index and religion also remain as significant factors in the Block 1 and adjusted models. We find that the effect of mother’s education is higher than the effect of father’s education (Table 2). The VIF values did not show any multicollinearity among the variables as all of those had values lower than 10 [33].

In Table 3, we present the findings from the mediation analysis. The direct effect of household food security is not significant on childhood anemia while the indirect effect of household food security mediating through maternal BMI and maternal anemia and childhood stunting is significant (*p* < 0.001). Maternal anemia and childhood stunting have significant direct and indirect effect on childhood anemia. The proportion of total effect of household food security passing through maternal BMI and maternal anemia and childhood stunting is 33%. The ratio of the indirect effect to direct effect of household food security is 0.5 which is half of the direct effect. The total effect of the household food security is 1.5 times the direct effect (Table 3).

**Table 3:** Result of the mediation analysis

Effect of the factors	Coefficient	SE	z	P	95% CI
Direct effect of household food security on childhood anemia (a path)	0.04	.025	1.61	0.11	−0.01 - 0.09
Direct effect of household food security on maternal anemia (d path)	0.06	.025	2.29	0.02*	0.01–0.11
Direct effect of household food security on maternal BMI (b path)	0.07	.025	2.62	0.01*	0.02–0.12
Direct effect of household food security on childhood stunting (f path)	0.1	0.03	3.94	0.00***	0.05–0.15
Indirect effect of household food security on childhood anemia	0.02	0.01	2.93	0.00***	0.01–0.03
Indirect effect of household food security on childhood anemia through maternal BMI (bc path)	0.02	0.02	0.71	0.48	−0.03-0.07
Indirect effect of household food security on childhood anemia through maternal anemia (de path)	0.16	0.02	6.59	0.00***	0.11–0.21
Indirect effect of household food security on childhood anemia through childhood stunting (fg path)	0.05	0.03	2.11	0.04*	0.00–0.10
Total effect of household food security on childhood anemia.	0.06	0.03	2.22	0.03*	0.01–0.11

*p < 0.05; **p < 0.01; ***p < 0.001

## Discussion

In this study, we estimated the socioeconomic determinants of anemia in children 6 to 59 months in Bangladesh with a special emphasis on household food security. The study reveals that maternal anemia is an important factor besides the age of children. We did not find any significant association between household food insecurity and childhood anemia while controlling other socioeconomic and demographic variables. However, food security was mediated by childhood stunting, maternal BMI and maternal anemia.

The percentage of anemia is very high at the age interval 6 to 23 months which is in line with other study [35] and then the prevalence declines. At 6 to 23 months of age, the requirement for iron is high as the growth and development of the child peaks at this stage [36]. Moreover, generally from 6 months, the children are introduced to the family food which, if lacking iron, makes the children more susceptible to become anemic. Along with the age, chronic undernutrition (stunting) of the children is significantly associated with childhood anemia which in line with several studies [37–39]. The argument is that most of the childhood undernutrition risk factors overlap such as poor socioeconomic status, suboptimal feeding, household food insecurity and poor hygiene practices together with mediocre access and utilization of health services may lead to co-occurrence of anemia and stunting.

We found that health status of the mother which include maternal anemia and BMI was highly associated with childhood anemia in Bangladesh, which is in line with prior studies [23–25, 40]. Maternal anemia works in several pathways. For instance, anemia at the time of pregnancy may contribute to low birth weight and preterm birth, both of which increase the risk of childhood anemia [41]. Maternal anemia may also reduce iron content in breast milk [42]. Children’s iron intake is low from breast milk and Bangladesh has a tradition of long duration of breastfeeding [15]. Moreover, mother and child share the same socioeconomic environment and when the child is on family food, the diet quality may be similar [40] indicating a concurrent development of anemia. The same argument goes for maternal BMI and studies from Bangladesh has shown that mothers with low BMI have the highest risk of having low birth weight baby and preterm baby [43].

It has been suggested that household food insecurity has a positive relationship with childhood anemia [40, 44]. However, the results of the current study do not support this relationship for Bangladesh while controlling for other socioeconomic and demographic variables. A possible explanation may be that in BDHS’2011, the food security measures only access to food and quantity of food. It does not capture the quality or the nutritional value of the food. It is possible that households deprived of iron-rich food such as meat and fish still was considered food secure. The nutritional quality of food is as important as the quantity of the food to term a household food secure. Other questionnaires or instruments to measure household food security have considered the nutritional quality of food [45] which was missing in the HFIAS. In Bangladesh, calories are predominantly from cereal-based foods, mainly rice, which contains inhibitors for iron absorption [40]. Therefore, the food security scale used in BDHS’11 i.e. HFIAS may not have captured the food security condition in terms of nutritional value. Furthermore, the dietary diversity of the Bangladeshi people is poor. Only 22.8% of the children aged 6–23 months are practicing optimal Infant and Young Child Feeding (IYCF) and around 59% of the women consumes fewer than five out of ten food groups which has not changed so much since 2005 [46].

Many studies find that the prevalence of anemia is higher in low-income families as suggested in a recent review [47], which was also the findings of the current study. Richer households can purchase more food, especially luxury food items (in the context of Bangladesh) such as meat and fish that are rich in iron.

Parents’ level of education have previously been shown to be an important determinant of childhood anemia [25] which is in line with our findings. One explanation is that parental education may affect childhood anemia through proximate determinants such as maternal health status and food security which play a more dominant role in childhood anemia than parental education [48]. Pashicha et al. stated that paternal education is underlying determinants of childhood anemia whereas maternal anemia is lifecycle determinants [49]. Moreover, the wealth index might have captured some of the effects of parental education as higher educated parents generally have higher wealth status. Due to collinearity, we use mother’s education and father’s education in two separate models (Block 1 & 2).

We also found some regional differences as living in Barisal, Khulna and Rangpur reduced the chance of not being anemia, compared to Sylhet. The possible reason may be the geographical differences in terms of source and production of food, infrastructure, and access to health care facilities. It is found that Barisal, Khulna and Rangpur divisions are poorer than other divisions which might be the reason for more anemic children in those particular regions, even though we control for household wealth status [50].

We also found that religion is a significant factor where Muslim children have a higher chance of not being anemic, compared to Hindu and other religions. Religion itself may not be the cause of the finding but may rather work through differences in dietary patterns. Hindu families eat more vegetarian diets compared to Muslim families which often contain less bioavailable iron [51]. Furthermore, Hindu families of Bangladesh never consume beef, which is a good source of iron, as their religion prohibit it [51] and beef is cheaper than other animal meat (e.g. mutton).

There are several other individual, household, and community characteristics that have the potential to affect childhood anemia as presented in a systematic literature review [19]. Some examples are parasitic infection, low birth weight, number of children ever born, birth interval, maternal autonomy on healthcare decision making, parental working status and household size. We could not control for parasitic infection and low birth weight in the analysis due to data unavailability. However, it can be argued that parasitic infection usually occurs in older children who are less prone to anemia and thus have less importance as a risk factors for anemia [52], and especially for the age group covered in the current study. Low birth weight could be an important factor as these children have lower storage of iron at time of birth. It is difficult in Bangladesh to know the birth weight as children usually are born in the homes, which is particular common in rural areas. The BDHS’2011 have information on size at birth which we used as a proxy of low birth weight. We have checked the remaining variables in our analyses and no statistical associations were found. The variables were dropped from the final models but presented in the supplementary material (Table S1). The BDHS’2011 has information on infant and young feeding practices (IYCF) which tell us on whether the diet for the children is diversified. However, IYCF only measures for children aged 6–23 months [15]. Therefore, we did not include this information in our study since the age of our sample is from 6 to 59 months.

The study has several strengths as well as limitations. It is based on the BDHS’2011, a representative national survey with large sample size, and the only one including the variables of interest for the current study, i.e., household food security, maternal anemia, and childhood anemia. The latest BDHS surveys, i.e., BDHS’14 and BDHS’18 did not have data on any of these variables, hence prevent us to perform analyses based on recent data. Although the data is relatively old, we believe that the research is still relevant. For example, according to the latest available data from the FAOSTAT, the number of moderately or severely food insecure people (3-year average) in 2014–2016 in Bangladesh was 50.4 million whereas in 2017–2019 it was 50.8 million [53]. And, the prevalence of anemia among women of reproductive age (15–49 years) was 41.4% in 2010 while in 2016 it was 39.9% [53]. Moreover, the Global Hunger Index showed that 13.8% of the Bangladeshi population was undernourished in 2010 whereas in 2020, the rate was 13% [12]. Therefore, over a decade a drastic change is not observed in Bangladesh in terms of anemia and household food security. The cross-sectional nature of the survey only describes association and not causality. However, this article is among the few that studied socioeconomic determinants of childhood anemia using national level data in Bangladesh.

The Bangladeshi Government should focus more on ensuring that the micronutrients are available to the mother and children under 5 years of age. Since rice and/or cereal-based products are the predominant diet of the Bangladeshi people, fortification of cereals with iron, micronutrient powder [54] can be a great help together with promotion of diversified diet to curb the burden of anemia. The Govt. has already taken these initiatives however, the coverage and progress are low [14]. The Government of Bangladesh has introduced micronutrient rich fortified rice through open market sale (OMS) where poor people can buy daily necessities with subsidies price [55]. The rice is fortified with Vitamin A, Vitamin B1, Vitamin B12, Folic acid, Iron and Zinc. This is a way to ensure that poor people who only eat rice can also get essential micronutrient, which may ensure food security and reduce the burden of childhood anemia. The National Strategy on Prevention and Control of Micronutrient Deficiencies of Bangladesh also introduced micronutrient powder (MNP) enriched with five nutrients including iron for the young children in few districts of Bangladesh [56]; but the full potential of MNP is still unknown [54]. However, political commitment and increasing awareness are required to ensure national level uptake of these initiatives.

## Conclusion

Anemia has been recognized as a major public health problem in Bangladesh for many years; still, the basic problem has not been solved but continues to negatively affect children as well as mothers. In the case of Bangladesh, preventing childhood anemia requires special attention to improved maternal health status before and during pregnancy as well as during the breastfeeding period. Other socioeconomic aspects do not have a direct effect on childhood anemia although for example improved economic status and food stuff variability could work in improving the mother’s health status and thus reduce childhood anemia levels. Further research is required to establish the casual pathways where household food security needs to be estimated with accounting for the quantity as well as the nutritional quality of the food for the women and children. Despite available effective ways to tackle anemia, the battle is yet far from won.

## Supplementary Information

**Additional file 1: Table S1.** Multivariate logistic regression analyses.

## Data Availability

The datasets for this study are available from the Demography and Health Survey (https://dhsprogram.com/data/dataset/Bangladesh_Standard-DHS_2011.cfm?flag=0) upon request. This data is freely available for the researchers.

## Abbreviations

BDHS: Bangladesh Demographic and Health Survey
BMI: Body mass index
CI: Confidence Interval
HFIAS: Household Food Insecurity Access Scale
NIPORT: National Institute for Population Research and Training
OR: Odds ratios
VIF: Variance Inflation Factor
