# Supplementary material for: Maternal health status and household food security on determining childhood anemia in Bangladesh -a nationwide cross-sectional study
Source: BMC Public Health. 2021 Aug 21;21:1581. doi: 10.1186/s12889-021-11581-3 (PMC8380337; doi:10.1186/s12889-021-11581-3)
Supplement: Supplementary file 1 — Additional file 1: Table S1. Multivariate logistic regression analyses. [file 12889_2021_11581_MOESM1_ESM.docx]

Table-S1: Multivariate logistic regression analyses

|  | **Adjusted odd ratio** |
| --- | --- |
| **Household Food Security** | |
| Food secure ® | 1 |
| Food insecure | 1.06 (0.79 - 1.42) |
| **Wealth index** | |
| Poorest | 1.15 (0.65 - 2.04) |
| Poorer | 1.18 (0.69 - 2.03) |
| Middle | 0.96 (0.57 - 1.62) |
| Richer | 0.88 (0.55 - 1.41) |
| Richest ® | 1 |
| **Religion** | |
| Muslim | 0.58** (0.36 - 0.95) |
| Hindu and others ® | 1 |
| **Region** | |
| Barisal | 1.93*** (1.20 - 3.11) |
| Chittagong | 1.03 (0.66 - 1.60) |
| Dhaka | 0.91 (0.59 - 1.40) |
| Khulna | 1.48 (0.92 - 2.40) |
| Rajshahi | 1.14 (0.71 - 1.82) |
| Rangpur | 1.78** (1.12 - 2.81) |
| Sylhet ® | 1 |
| **Father’s education** | |
| No or low education | 1.34 (0.94 - 1.91) |
| High education ® | 1 |
| **Mother’s BMI** | |
| Under/over weight | 1.16 (0.88 - 1.54) |
| Normal ® | 1 |
| **Mother’s anemia** | |
| Anemic | 2.03*** (1.56 - 2.65) |
| Not anemic ® | 1 |
| **Mother’s education** | |
| No or low education | 1.93* (0.99 - 3.75) |
| High education ® | 1 |
| **Sex of children** | |
| Male | 1.34** (1.02 - 1.75) |
| Female ® | 1 |
| **Age of the children** | |
| 6-12 | 3.74*** (2.25 - 6.21) |
| 13-24 | 3.78*** (2.41 - 5.93) |
| 25-36 | 1.86*** (1.24 - 2.78) |
| 37-48 | 1.37* (0.96 - 1.96) |
| 49-59 | 1 |
| **Currently Breastfeeding** | |
| Yes | 0.97 (0.69 - 1.35) |
| No ® | 1 |
| **Child Nutritional status** | |
| Stunted | 1.32** (1.01 - 1.73) |
| Normal ® | 1 |
| **Birth Size** | |
| Large | 1.01 (0.70 - 1.47) |
| Small | 1.01 (0.72 - 1.41) |
| Average® | 1 |
| **Household size** | |
| > 6 | 1.21 (0.83 - 1.77) |
| 5 to 6 | 1.27 (0.84 - 1.91) |
| < 5® | 1 |
| **Total children** | |
| 1-2 child ® | 1 |
| more than 2 child | 1.05 (0.76 - 1.45) |
| **Birth interval** | |
| >= 24 months | 1.32 (0.85 - 2.04) |
| 9-23 months ® | 1 |
| **Decision on health care** | |
| Together (Husband and Wife) | 1.13 (0.74 - 1.73) |
| Husband and other | 1.24 (0.80 - 1.92) |
| Alone ® | 1 |
| **Father's occupation** | |
| Low income | 0.76 (0.47 - 1.24) |
| Middle income | 0.80 (0.49 - 1.31) |
| Rich income ® | 1 |
| **Residence** | |
| Urban ® | 1 |
| Rural | 1.16 (0.82 - 1.63) |
| *p<0.05 ; **p<0.01; ***p<0.001 | |
